# Atypical Chest Pain Revealing a Coronary Artery Dissection on an Atherosclerotic Plaque

**DOI:** 10.7759/cureus.102394

**Published:** 2026-01-27

**Authors:** Youssef Daoudi, Hibat Allah Kamri, Lebbar Samy, Fatimazahra Merzouk, El Ghali Mohamed Benouna

**Affiliations:** 1 Cardiology, Cheikh Khalifa International University Hospital – Mohammed VI University of Health Sciences (UM6SS), Casablanca, MAR; 2 Cardiology, Mohammed VI International University Hospital – Mohammed VI University of Health Sciences (UM6SS), Casablanca, MAR

**Keywords:** acute coronary syndrome (acs), case report, male, percutaneous coronary intervention (pci), sudden atherosclerotic coronary artery dissection

## Abstract

Coronary artery dissection overlying atherosclerotic plaque represents a rare but important mechanism of acute coronary syndrome (ACS). It differs from spontaneous coronary artery dissection (SCAD), which typically occurs in angiographically normal arteries. Recognizing this distinction is critical for optimal management and reporting.

We report the case of a 53-year-old diabetic male who presented with atypical chest pain. ECG showed nonspecific repolarization changes, and troponins were mildly elevated. Coronary angiography, performed 12 hours after symptom onset, revealed a significant stenosis and dissection at the bifurcation of the mid-left anterior descending (LAD) artery and diagonal branch. Atherosclerotic plaque was evident at the dissection site. No intravascular imaging was available to further confirm plaque disruption. The patient was treated with a provisional stenting strategy using a drug-eluting stent (DES), with excellent angiographic and clinical outcomes.

Coronary artery dissection occurring on a vulnerable atherosclerotic plaque is a rare but clinically relevant cause of ACS. Differentiation from SCAD is essential, as it has distinct pathophysiology, prognosis, and therapeutic implications. This case highlights the need for refined classification systems and dedicated guidelines addressing plaque-related dissections.

## Introduction

Coronary artery dissection is a relatively rare but clinically significant cause of acute coronary syndrome (ACS). Among its forms, spontaneous coronary artery dissection (SCAD) is increasingly recognized, particularly in women without traditional cardiovascular risk factors. In contrast, dissection occurring in the presence of atherosclerotic disease is a distinct and underrepresented entity in the literature.

Dissection on an atherosclerotic plaque typically arises from plaque rupture, erosion, or intraplaque hemorrhage, leading to intimal tearing and intramural hematoma formation. While this mechanism is acknowledged in the general ACS literature, it is not emphasized in SCAD-focused consensus statements. The 2020 AHA SCAD scientific statement does not include detailed management considerations for plaque-related dissections [1].

Angiographically, these lesions may mimic SCAD, particularly the Type 2 pattern characterized by long, smooth, tapering stenosis. However, applying the SCAD classification to atherosclerotic dissections has limitations, as these dissections do not occur in healthy vessels and differ in prognosis and recurrence risk. While SCAD has a high rate of spontaneous healing and low recurrence in males, plaque-related dissections carry an underlying risk of progressive atherosclerosis and recurrent ACS and require more aggressive management [2,3].

Published data estimate that SCAD accounts for 1-4% of ACS presentations overall, rising to 25-30% in women under 50 [1]. In contrast, plaque-associated dissections are not well quantified but are likely underreported due to misclassification. Their recognition is clinically relevant, as management diverges: SCAD is often treated conservatively, while flow-limiting plaque dissections generally require revascularization [2,4].

This case contributes to bridging the gap between SCAD literature and broader ACS understanding by detailing angiographic and clinical clues to distinguish plaque-related dissection. It also highlights the absence of clear diagnostic or management guidelines specific to this entity, reinforcing the need for future studies and reporting.

## Case presentation

A 53-year-old man with a past medical history significant for poorly controlled type 2 diabetes mellitus and prior thyroidectomy with stable hormone replacement therapy presented with chest discomfort of atypical characteristics. He had no known history of coronary artery disease.

The patient reported the recent onset of intermittent, left-sided chest discomfort, described as a pressing sensation. The pain episodes lasted several minutes and were not associated with dyspnea, palpitations, or diaphoresis. The patient was hemodynamically stable, with a blood pressure of 135/82 mmHg and a heart rate of 78 bpm. Physical examination showed no abnormalities, with normal peripheral pulses and no signs of heart failure.

**Figure 1:**
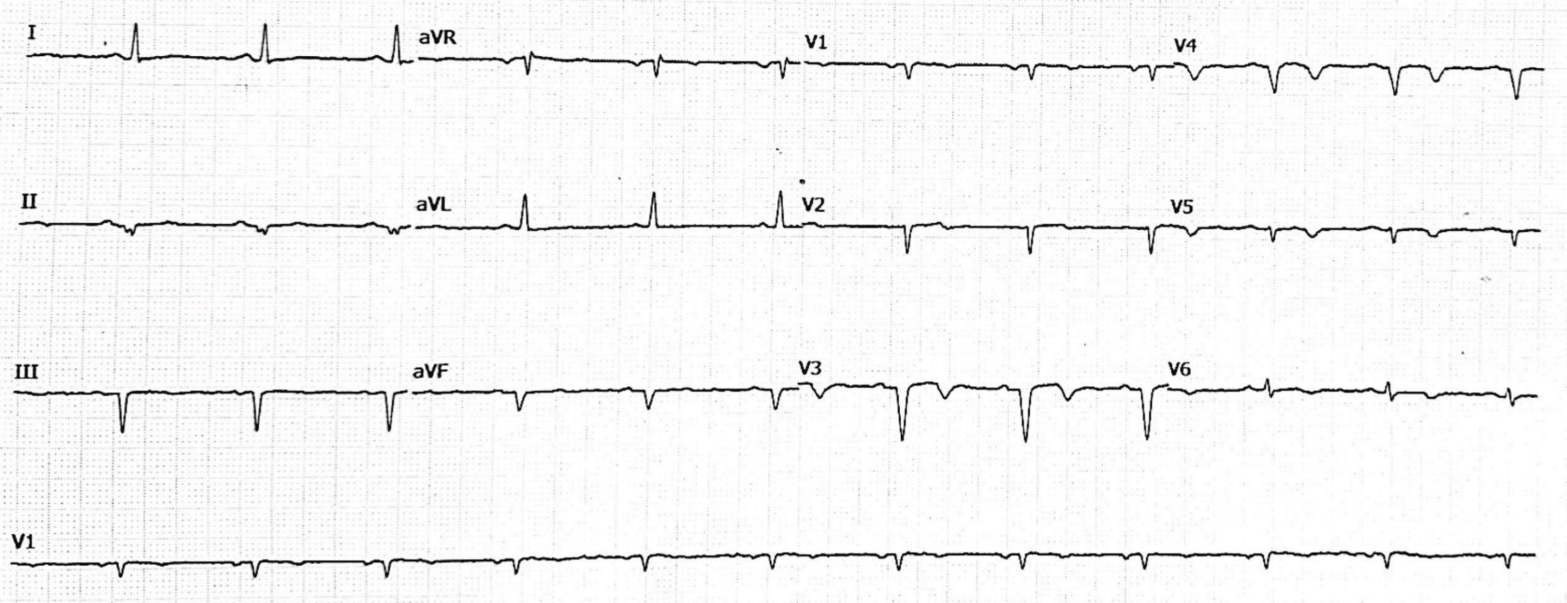
Resting 12-lead electrocardiogram Resting 12-lead electrocardiogram: sinus rhythm, left axis deviation, and diffuse T-wave inversions in the anterior and lateral leads, consistent with nonspecific repolarization abnormalities suggestive of myocardial ischemia.

An ECG was performed and showed T-wave inversions in the anterior and lateral leads (Figure 1). High-sensitivity troponin was mildly elevated (peak at 0.12 ng/mL), suggesting a non-ST elevation myocardial infarction (NSTEMI).

Additional laboratory workup revealed elevated low-density lipoprotein (LDL) cholesterol at 142 mg/dL, low high-density lipoprotein (HDL) cholesterol at 38 mg/dL, and a total cholesterol level of 215 mg/dL, along with an HbA1c of 8%, indicating suboptimal glycemic control.

**Figure 2:**
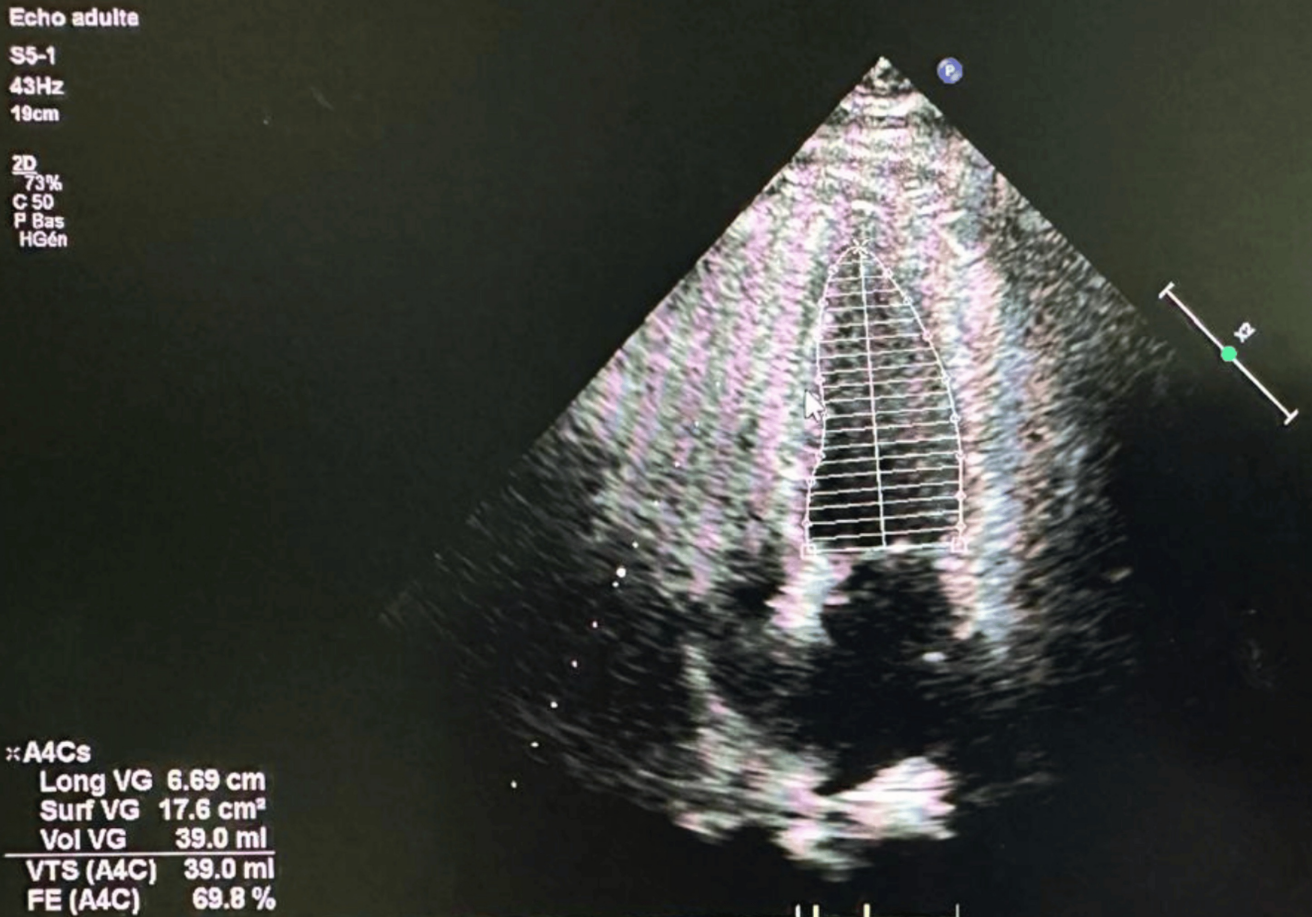
Transthoracic echocardiographic apical four-chamber view showing preserved left ventricular systolic function.

Transthoracic echocardiogram found a preserved left ventricular systolic function with no regional wall motion abnormalities (Figure 2).

**Figure 3:**
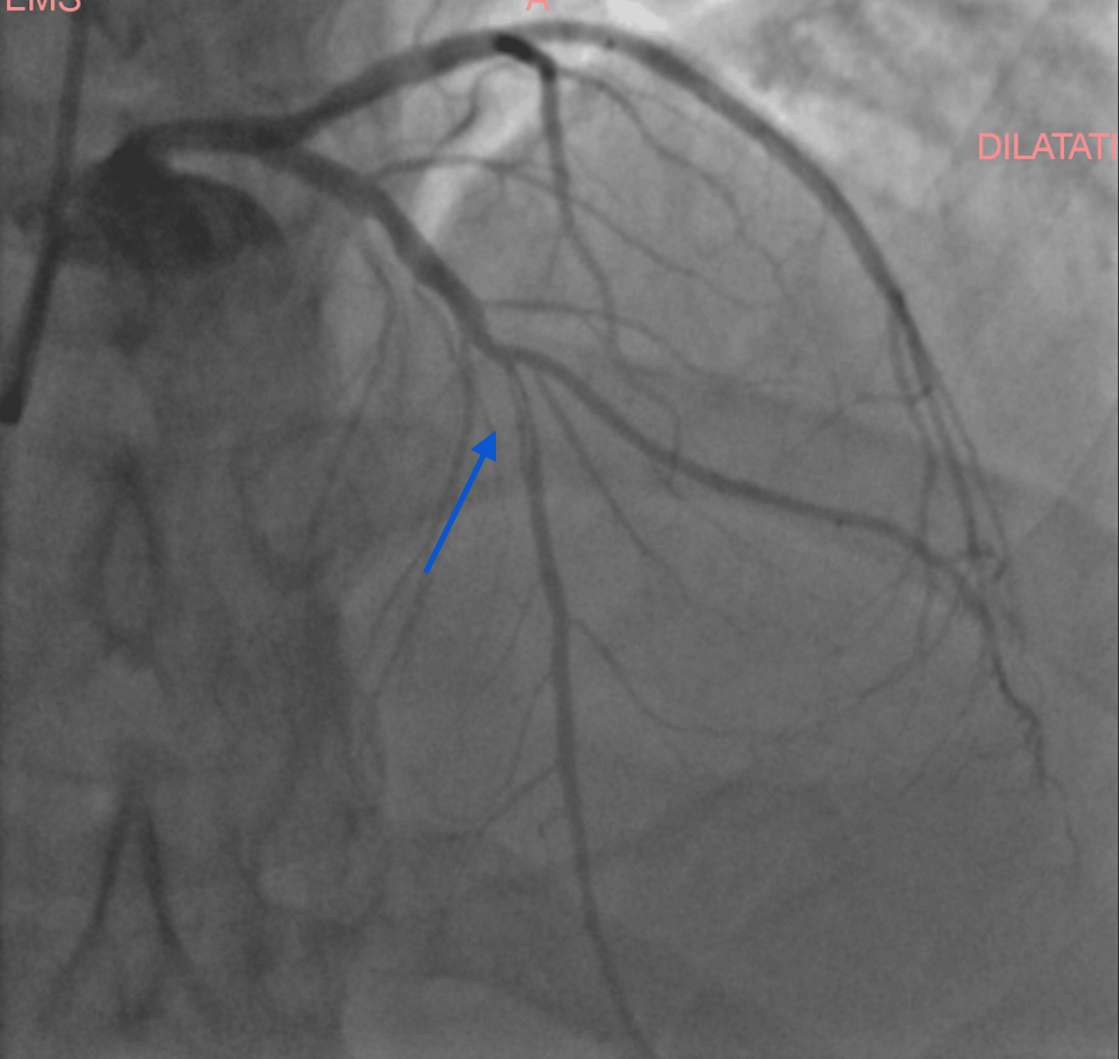
Coronary angiographic image of the patient Coronary angiographic image coronary artery dissection of the middle section of the left anterior descending artery (arrow), involving the bifurcation with the diagonal branch. The lesion appears as a long, smooth narrowing typical of Type 2.

The angiography revealed a significant stenosis at the bifurcation of the middle section of the left anterior artery and diagonal branch, with angiographic features highly suggestive of coronary artery dissection involving the mid-LAD. The dissection presented as a long, smooth narrowing with contrast staining, consistent with a Type 2 coronary artery dissection pattern (Figure 3). Intravascular imaging with OCT or IVUS was considered but ultimately not performed, due to lack of availability at the time of intervention.

**Figure 4:**
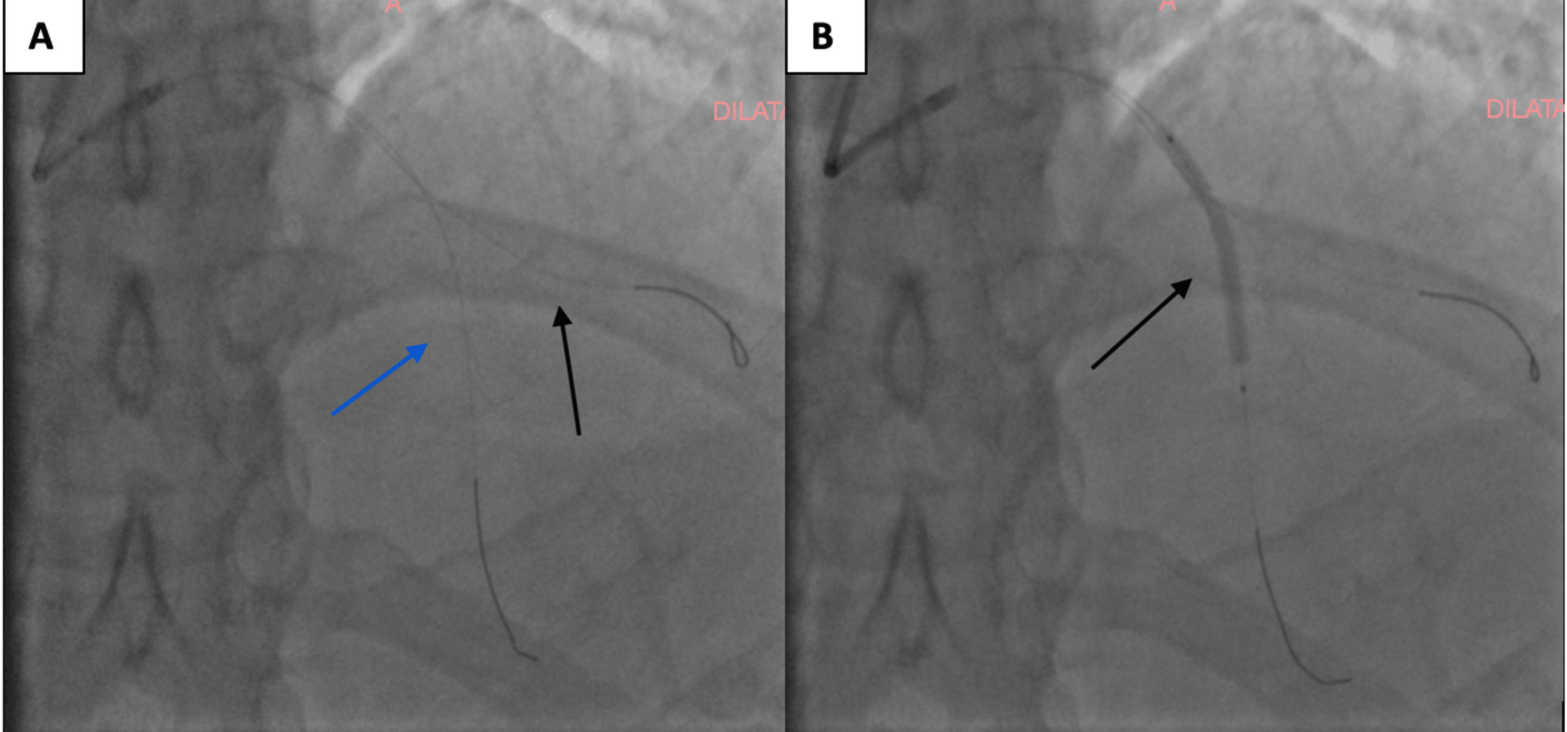
Provisional stenting strategy. (A) Initial wiring of both the left anterior descending artery (black) and the diagonal branch (blue arrow) using two separate guidewires. This step ensures protection of the side branch before stent deployment in the LAD. (B) Deployment of the stent (black arrow) in the middle section of the LAD across the bifurcation.

A provisional stenting strategy was selected, aiming to treat the LAD while protecting the diagonal branch (Figure 4).

**Figure 5:**
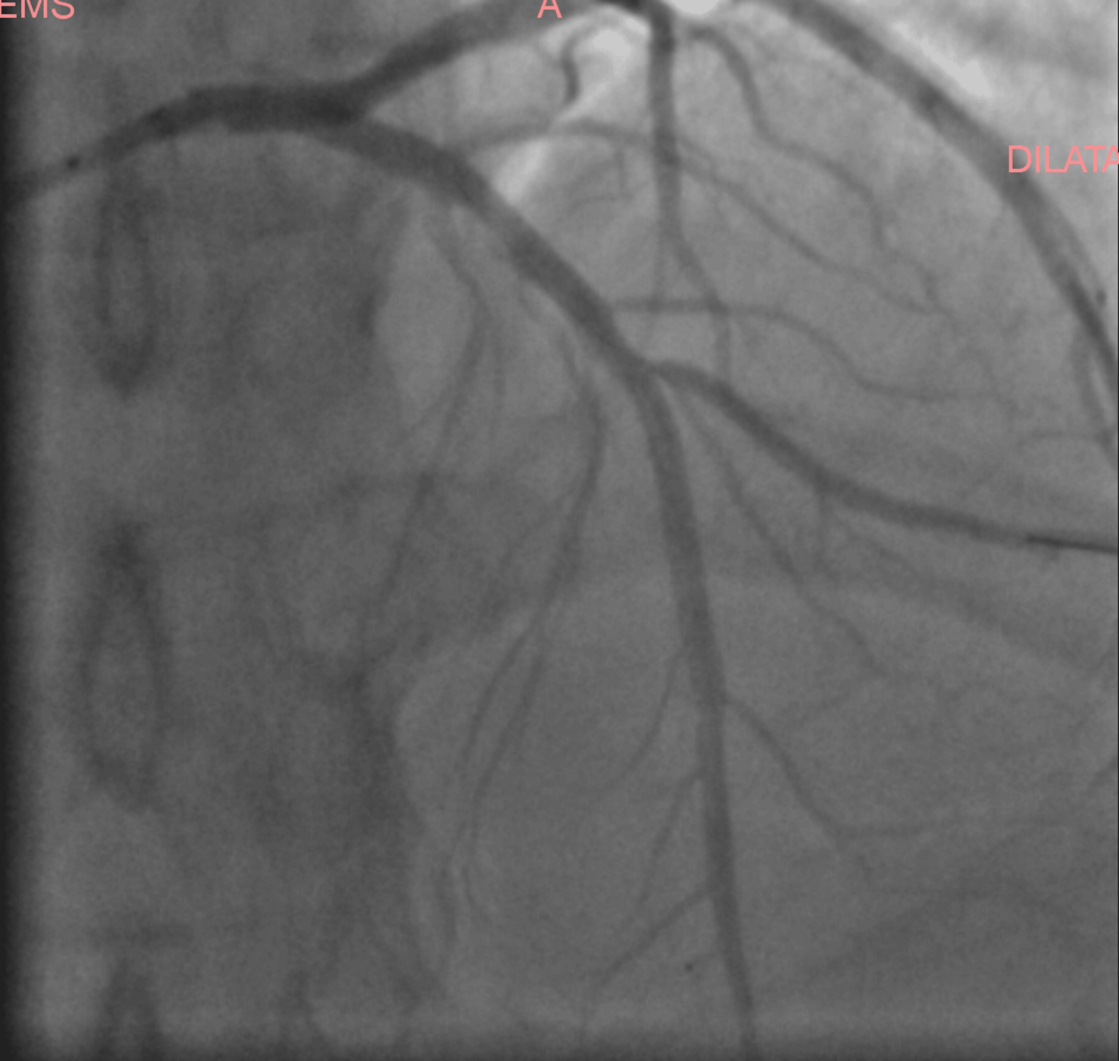
Final coronary angiogram following percutaneous coronary intervention with stent implantation in the mid-left anterior descending (LAD) artery. The result shows excellent stent expansion, restoration of vessel patency, and preserved flow in the diagonal branch (TIMI 3 flow).

Under radial artery access, a drug‑eluting stent was deployed in the proximal LAD with restoration of normal luminal contour and TIMI 3 flow. The procedure was uncomplicated (Figure 5).

The patient remained hemodynamically stable postprocedure and was discharged the following day on dual antiplatelet therapy (aspirin + clopidogrel), high‑intensity statin, ACE inhibitor, and lifestyle modification advice. Follow‑up at one and three months demonstrated the absence of symptoms and good functional capacity.

## Discussion

Dissection of a coronary artery overlying an atherosclerotic plaque represents an uncommon yet clinically significant cause of ACS. It is a pathophysiological entity distinct from SCAD, which typically occurs in angiographically normal arteries, in young to middle-aged women, and in the absence of traditional cardiovascular risk factors. In contrast, dissections associated with atherosclerotic disease generally occur in older individuals with risk factors such as hypertension, diabetes, and dyslipidemia, as in our patient [1,2].

Plaque-related coronary artery dissection involves structural disruption of the arterial wall, often initiated by rupture or fissuring of a vulnerable atherosclerotic plaque. This event may lead to the formation of a false lumen or intramural hematoma. Another proposed mechanism is rupture of the vasa vasorum within the atherosclerotic segment, leading to intramural hemorrhage and dissection, even in the absence of plaque rupture [2]. These processes can create a spiral dissection and compromise coronary flow, leading to myocardial ischemia or infarction [3].

Although SCAD has been extensively studied and described in recent years, the literature on dissections associated with plaque is relatively scarce. In a study by Daoulah et al., plaque-related dissections were predominantly observed in male patients and were associated with more extensive atherosclerotic disease and a higher need for revascularization, compared with SCAD [1]. These findings highlight the importance of distinguishing between these two entities, especially in terms of management and prognosis.

Optical coherence tomography and intravascular ultrasound are essential for distinguishing between SCAD and plaque-related dissection, especially in angiographically ambiguous cases. These modalities can visualize features such as plaque rupture, thin fibrous caps, lipid-rich cores, or intramural hematomas, thus confirming the underlying mechanism [4]. Unfortunately, these tools were not available in our case, but the presence of an angiographically visible plaque at the dissection site strongly supported the diagnosis of dissection secondary to plaque disruption.

Management strategies differ significantly between SCAD and plaque-related dissections. SCAD is often treated conservatively when the patient is stable and the lesion is nonobstructive, as the artery frequently heals spontaneously. In the context of plaque-related dissection, particularly when the dissection is flow-limiting, involves a bifurcation, or is associated with ongoing ischemia, revascularization is generally required. In a study by Lettieri et al., PCI was shown to be both safe and effective in patients with atherosclerosis-related dissections, supporting early intervention in such cases [5]. In our case, given the involvement of the LAD-D1 bifurcation and the hemodynamic significance of the lesion, percutaneous coronary intervention (PCI) with a provisional stenting strategy was appropriate and led to an excellent angiographic and clinical outcome.

Although current ACS and SCAD consensus guidelines primarily focus on nonatherosclerotic spontaneous dissections, they do acknowledge the existence of plaque-related dissections as a distinct mechanism requiring tailored management. The "Type 2" angiographic pattern observed in this case, characterized by a long, smooth tapering, derives from SCAD classifications. However, this framework may not fully apply to atherosclerotic dissections, which occur in different patient populations and involve distinct pathophysiological mechanisms. While SCAD often follows a benign course when managed conservatively, plaque-associated dissections carry a higher risk of recurrent ischemia and typically warrant revascularization, with implications for long-term outcomes and secondary prevention.

This case highlights the diagnostic and therapeutic challenges in managing coronary artery dissections associated with atherosclerotic plaques. The importance of distinguishing these lesions from SCAD lies not only in their pathophysiology but also in their management, as the need for early intervention is more frequent and crucial in plaque-related dissections.

## Conclusions

This case illustrates a coronary artery dissection occurring on a pre-existing atherosclerotic plaque in a middle-aged male with diabetes. Accurate recognition of this distinct mechanism is essential, as it impacts therapeutic strategy and prognosis. PCI using provisional stenting in bifurcation lesions can be safe and effective in this context.

Heightened clinical awareness of this presentation may support earlier recognition and appropriate intervention in similar cases. This case adds to the limited body of literature on atherosclerotic plaque-related coronary dissections, particularly in middle-aged diabetic patients, and emphasizes the need for clearer diagnostic criteria and tailored management strategies. Future reports and registries focusing specifically on plaque-associated dissections may help refine therapeutic approaches and improve prognostication.
